# Legionellosis Associated with Recreational Waters: A Systematic Review of Cases and Outbreaks in Swimming Pools, Spa Pools, and Similar Environments

**DOI:** 10.3390/ijerph15081612

**Published:** 2018-07-30

**Authors:** Erica Leoni, Federica Catalani, Sofia Marini, Laura Dallolio

**Affiliations:** 1 Unit of Hygiene, Public Health and Medical Statistics, Department of Biomedical and Neuromotor Sciences, University of Bologna, via S. Giacomo 12, 40126 Bologna, Italy; laura.dallolio@unibo.it; 2 School of Hygiene and Preventive Medicine, Department of Biomedical and Neuromotor Sciences, University of Bologna, via S. Giacomo 12, 40126 Bologna, Italy; federica.catalani@studio.unibo.it; 3 Department of Life Quality Studies, University of Bologna, Campus of Rimini; Corso d’Augusto 237, 47921 Rimini, Italy; sofia.marini2@unibo.it

**Keywords:** *Legionella* spp., Legionnaires’ disease, Pontiac fever, recreational water, hot tubs, whirlpools, spa pools, swimming pools

## Abstract

*Legionella* spp. is widespread in many natural and artificial water systems, such as hot water distribution networks, cooling towers, and spas. A particular risk factor has been identified in the use of whirlpools and hot tubs in spa facilities and public baths. However, there has been no systematic synthesis of the published literature reporting legionellosis cases or outbreaks related to swimming/spa pools or similar environments used for recreational purposes (hot springs, hot tubs, whirlpools, natural spas). This study presents the results of a systematic review of the literature on cases and outbreaks associated with these environments. Data were extracted from 47 articles, including 42 events (17 sporadic cases and 25 outbreaks) and 1079 cases, 57.5% of which were diagnosed as Pontiac fever, without any deaths, and 42.5% were of Legionnaires’ disease, with a fatality rate of 6.3%. The results are presented in relation to the distribution of *Legionella* species involved in the events, clinical manifestations and diagnosis, predisposing conditions in the patients, favourable environmental factors, and quality of the epidemiological investigation, as well as in relation to the different types of recreational water sources involved. Based on the epidemiological and microbiological criteria, the strength of evidence linking a case/outbreak of legionellosis with a recreational water system was classified as strong, probable, and possible; in more than half of the events the resulting association was strong.

## 1. Introduction

Legionellosis is a disease transmitted through the inhalation of particles of aerosolized water contaminated by the opportunistic waterborne pathogen, *Legionella* spp. [1]. After the first recognition of legionellosis in 1976, when 221 participants of the annual convention of the American Legion contracted pneumonia and 34 of them died, surveillance systems were developed and implemented in several countries [2]. Legionellosis surveillance is a current public objective: In 2015, according to the European Centre for Disease Prevention and Control surveillance, 7034 cases were reported in Europe, concerning 1.4 cases per 100,000 inhabitants [3].

The majority of outbreaks described in the literature are correlated to *Legionella pneumophila*, in particular serogroup 1, but other serogroups and species were also associated to human disease, such as *L. micdadei* (now classified as *Tatlockia micdadei*), *L. dumoffii*, and *L. longbeachae* [4]. The two fundamental clinical pictures determined by these infective agents are Legionnaires’ disease (LD) and Pontiac fever (PF): The former is generally characterized by an acute pneumonia and, rarely, by an extrapulmonary disease; Pontiac fever is a mild, self-limiting, flu-like illness, which resolves in a few days.

*Legionella* spp. are widely distributed in both natural (i.e., lakes, rivers, groundwater, thermal water) and man-made aquatic environments, such as the water systems of hospitals, hotels, private houses [5,6], cooling towers [7], dental units [8,9], and recreational [10,11] or therapeutic [12,13] facilities. Any system or equipment which contains, stores, or re-circulates non-sterile water that can be aerosolized is a source of legionellosis [14,15]. Considering these elements, the recreational use of water is an important potential way of exposure to *Legionella* spp., especially in hot water pools equipped with hydromassage systems. A recent review on outbreaks of LD and PF highlights that 14% of the reported outbreaks from 2006 to 2017 recognized pools or spas as an attributed or suspected source [16]. The role of these recreational facilities appears even more significant if one considers the growing popularity of private hot tubs and the increasing number of people frequenting public spa pools and similar environments.

Generally, the outbreak analysis and control measures, specific for each exposure setting, are essential tasks of Public Health Authorities, including outbreak surveillance and analysis specifically dedicated to the recreational water context. Epidemiological knowledge about these themes must be constantly updated. To our knowledge, no systematic synthesis or critical appraisal exists of the published literature reporting sporadic cases or outbreaks of LD and/or PF associated with recreational water. In the present study, we performed a systematic review and analysis of investigations on legionellosis cases or outbreaks related to treated and untreated recreational water, including natural waters, swimming pools, spa pools, and similar environments (hot tubs, whirlpools, hot spring baths, etc.), in accordance with the definitions given for these environments by World Health Organization (WHO) guidelines [17].

## 2. Materials and Methods

In line with the objective of the study, we set out to perform a systematic review of cases and outbreaks of LD and PF associated with recreational aquatic environments, such as swimming and spa pools or natural spas. The literature search was conducted in Medline, including publications from 1 January 1977 (since the disease was first described in 1976) to 31 May 2018, using the following search terms: (Legionella OR legionellosis OR “Pontiac fever” OR “Legionnaires’ disease”) AND (case* OR cluster* OR outbreak* OR infection* OR investigation OR surveillance) AND (“recreational water” OR spa OR pool OR “swimming pool” OR “hot tub” OR whirlpool OR bath OR “swim spa” OR “turkish bath” OR sauna OR Jacuzzi OR “natural spa” OR “hot spring” OR “thermal spring” OR “warm spring” OR spring OR thermal). The literature search was conducted without language restrictions, on the condition that the articles had an exhaustive abstract in English reporting the information of interest. A further selection of relevant publications was performed using the inclusion and exclusion criteria listed below.

Inclusion criteria:

- Primary studies describing cases/outbreaks of LD or PF originating from recreational water.

Exclusion criteria:

- Not recreational water (hot water system, cooling tower, fountain, network water, therapeutic water, water births);
- environmental studies without cases;
- not primary studies;
- articles focused only on clinical and laboratory aspects;
- abstract not available/ not complete or not exhaustive;
- articles focused on pools used for display only (retail premises, fairs, exhibitions, shows);
- articles evaluating only microbiological risk assessment; and
- hot tubs or pools on cruise ships (due to a recently published systematic review) [18].

Two researchers independently screened titles and abstracts to identify potentially relevant articles and to exclude articles incompatible with the first five exclusion criteria; any disagreements were resolved by discussion with a third author. After the application of the first five exclusion criteria, the full texts of the remaining articles were examined, and any publications exclusively focused on display spas were then excluded, since this type of exposure in environments used for retail premises, fairs, exhibitions, and shows is not directly linked to recreational use. The remaining articles were assigned to three categories related to three different recreational facilities or sources of infection:

(a) Private hot tub and similar facilities;
(b) public pools and spas and similar facilities, generally supplied by municipal network water; and
(c) spa facilities supplied by natural water, or hot spring/thermal water. Subsequently, we applied the last two exclusion criteria to each category.

Data extracted from these publications included: Year, country, case definition, clinical form, type of event (sporadic case or outbreak), number of cases, attack rate, number of hospitalizations and/or deaths, risk factors, laboratory diagnosis, *Legionella* spp. involved, environmental isolates and concentrations (cfu/L), type of recreational water, water supply, and the type of epidemiological study carried out (descriptive, analytical, presence/absence of environmental investigation). An event with multiple cases (at least two) linked in space and time, with a suspected common source, was defined as an outbreak. For each event (both sporadic cases and outbreaks), epidemiological and microbiological criteria were adopted to characterize the strength of evidence linking the legionellosis event with the suspected recreational water system. Table 1 summarizes these criteria.

Data were analysed as the frequency distribution of the different variables included.

## 3. Results

**Figure 1:**
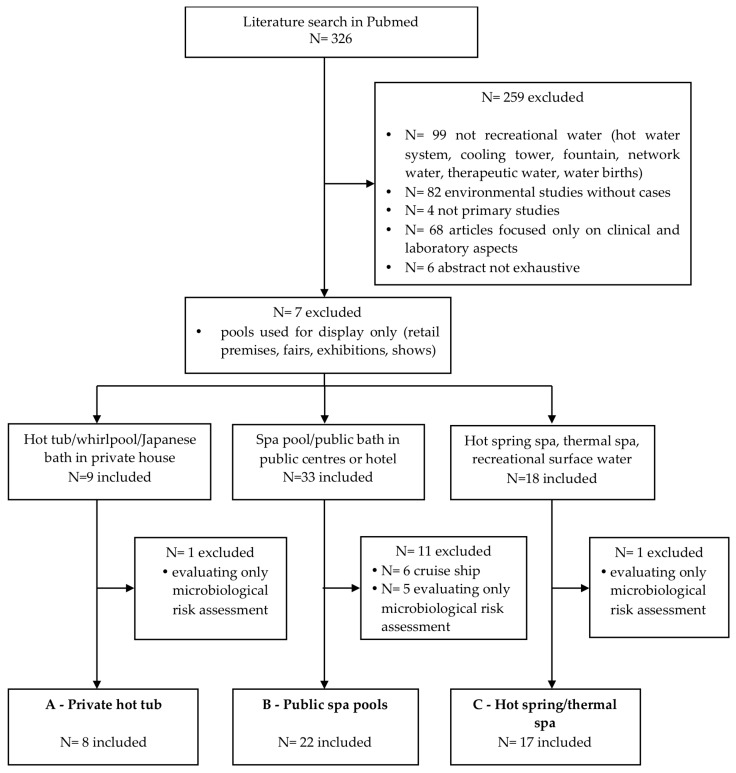
Flow chart of the selection process of articles.

Of the 326 articles retrieved from Medline, 259 were excluded for the following reasons: 99 investigations did not refer to recreational water, 82 were environmental studies without cases, 4 were not primary studies, 68 articles were focused only on clinical and laboratory aspects, and 6 publications were in a language other than English and did not have an exhaustive English abstract, as shown in Figure 1.

**Figure 2:**
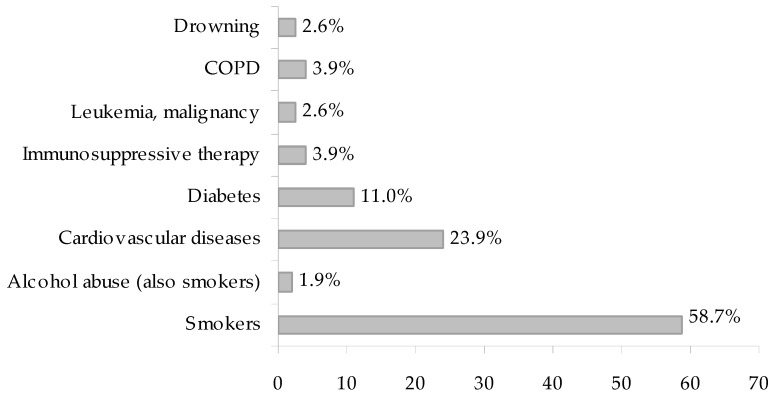
Distribution of underlying medical conditions and risk factors in 155 cases of Legionnaires’ disease.

At the end of the selection process, 47 articles were considered eligible for inclusion in the present review, corresponding to 42 events. In four cases, different articles described varying aspects of the same event, while two articles reported two and three different events, respectively. Among the 42 events of legionellosis, eight were linked to a hot tub/whirlpool/Japanese bath used in private houses (Category A in Figure 1, in brief “private hot tub”), 22 were related to whirlpool spa/baths in public centres and hotels (Category B in Figure 2, in brief “public spa pools”), and 12 to hot spring/thermal spa pools (Category C in Figure 1, in brief “hot spring/thermal spa”).

The selected articles were published: Four in the 1980s, 16 in the 1990s, 19 in the 2000s, and three from 2010 to 2018. In 11 articles, the authors did not report the date of onset. The events occurred in different countries across the world, with the highest frequency of hot spring related events in Japan (83.3%) and an overall highest frequency in Japan (18 events: 42.9%), followed by the USA (11 events: 26.2%), and the United Kingdom (4 events: 9.5%).

### 3.1. Legionellosis in Relation to Recreational Water Source

Table 2 shows all events and cases of legionellosis associated with recreational water systems, distinguished per facility category. Of the 1079 total cases included in the 42 events, 57.5% were diagnosed as PF, without any deaths, and 42.5% were of LD, with a fatality rate of 6.3%. 

The private hot tubs were all supplied by municipal network water and were subjected to a supplementary disinfection system only in two of the eight facilities involved in the legionellosis events. Single cases occurred in five events (62.5%) corresponding to 17.9% of cases, while the remaining three events were outbreaks with a low number of persons involved (from four to 13). LD represented 21.4% of the cases, with a fatality rate of 16.7%.

Public spa pools were generally supplied by municipal network water and only three out of 22 facilities had their own supply system from groundwater (two spa pools) and mountain spring water (one spa pool). In 54.5% of the facilities, water treatment included recycling, filtering, and chemical disinfection with bromine (seven spa pools) or chlorine (five spa pools). In the remaining public spa pools, water disinfection was not mentioned. Public spa pools were responsible for the highest number of events (22), cases (744), and deaths (16). A sporadic case only occurred in 9.1% of the events, while the remaining events were outbreaks often involving a high number of cases of up to 170 [19]. The LD cases formed 19.6% of the total cases, with a fatality rate of 11.0%.

Hot spring/thermal spas were supplied by natural waters, i.e., hot springs and thermal waters. This group also includes the only LD case associated with bathing in surface water. This was a fatal case in a 27-year-old woman who had nearly drowned in estuarine water [20]. Water treatment and chlorine disinfection were reported in only three out of the 11 hot spring/thermal water facilities (27.3%), while, in one case, the authors specified that national regulations (France) precluded the addition of chemicals to thermal spas to preserve the characteristics of the mineral water [21]. All cases linked to this recreational water category were diagnosed as LD, with a fatality rate of 3.9%. Single cases occurred in 83.3% of the events and only two outbreaks were reported. However, one of these was the largest outbreak of LD associated with a hot spring bathhouse in Japan, with 295 cases, including confirmed and probable cases [22].

### 3.2. Epidemiological Investigations

All the events with sporadic cases were studied by descriptive epidemiology. The epidemiological investigations included an analytical study in 36.0% of outbreaks, with higher percentages in events linked to public spa pools (40.0%) and hot spring/thermal water (50%), compared to private hot tubs (no events with an analytical study). An environmental investigation was carried out in 83.3% of events (private hot tubs and hot spring/thermal water: 75%; public spa pools: 90.9%) and allowed the detection of *Legionella* spp. in 76.2% of the incriminated water sources and to evidence identical molecular profiles of both clinical and environmental isolates in 33.3% of the events. Based on the epidemiological and microbiological criteria specified in Table 1, the strength of evidence linking the case/outbreak of legionellosis with the recreational water system was strong in 23 events (52.4%), with percentages higher for public spa pools (68.2%) and hot spring/thermal water (58.3%) compared to private hot tubs (12.5%). This was a consequence of the previously mentioned differences regarding both the implementation of analytic epidemiology and the detection of environmental *Legionella* spp., which were carried out less frequently in private hot tub related events.

### 3.3. Events with Sporadic Cases of Legionellosis

Sporadic cases of legionellosis occurred in 17 distinct events, only one of PF [23] and 16 of LD (Table 3), with a fatality rate of 29.4% (31.2% for LD cases). Most cases occurred in Japan (70.6%) [24,25,26,27,28,29,30,31,32,33,34,35], and hot spring/thermal waters (56.2%) were the facilities most involved, followed by private hot tubs (25%). Only two cases occurred in spa centres/public baths [35,36]. Four cases, three of which fatal, were consequent to near drowning [20,32,35,37] and one case involved a 10-year-old girl, subjected to immunosuppressive therapy for hemosiderosis after being exposed several times to the hot tub in her maternal home [38].

Etiological diagnosis was confirmed by culture of clinical specimens in 75.0% of LD cases and *L. pneumophila* was the species most frequently involved, in particular *L. pneumophila* SG 6 (31.2% of LD cases). No differences were observed on the onset of cases in relation to the different concentrations of legionellae detected from the suspected water sources. Genotyping of clinical and environmental isolates was performed in seven out of 17 events. In accordance with the microbiological criteria specified in Table 1, the strength of evidence linking the cases with the recreational water system was strong in all the cases confirmed by molecular typing (43.7% of LD cases).

### 3.4. Outbreaks of Legionellosis

A total of 25 outbreaks of legionellosis were found: 7 outbreaks of PF (Table 4), 11 outbreaks of LD (Table 5), and 7 mixed events of PF and LD (Table 6). Among the LD events, two were repeated cases on the same site, which occurred in different time periods (No. 2, 3 in Table 6), and one was a long-lasting outbreak with three consecutive clusters (No. 10 in Table 6). 

The total number of outbreak cases was 1062, of which 619 were PF cases (58.3%) and 443 were LD cases (41.7%), with 24 deaths (total fatality rate: 2.3%, for LD: 5.4%). Most events occurred in public spas (20/25 outbreaks, 80%), particularly in whirlpool spas of hotels or similar residential facilities, such as inns and holiday resorts (11 of 25 outbreaks, 44%). The attack rate varied from 29.8% to 86.7% for PF outbreaks and from 0.13% to 1.9% for LD outbreaks.

Etiological diagnosis was confirmed by culture of clinical specimens in 10 out of 11 outbreaks of LD and in one out of seven mixed events of PF and LD (61.1% of total events with LD cases), while it was never performed in PF outbreaks. *L. pneumophila* was the species most frequently involved, in particular *L. pneumophila* SG 1 in 68% of total outbreaks (83.3% of outbreaks with LD cases) and SG 6 in 24% of total outbreaks (27.8% of outbreaks with LD cases). In three events, various species or serogroups were identified as responsible for the disease by culture and/or serological assay. 

Environmental isolates of *Legionella* spp. were obtained in 22 outbreaks (88%), in seven of which various species or serogroups were detected (28%). Genotyping of clinical and environmental isolates was performed in 10 events (40% of total outbreaks, 55.5% of outbreaks with LD cases). In accordance with the epidemiological and microbiological criteria specified in Table 1, the strength of evidence linking the outbreak with the recreational water system was strong in 16 events (64%).

### 3.5. Patient Contributing Factors

PF cases showed no evidence of underlying risk factors. The median age of the PF patients, when reported, varied from 12 to 54 years and, overall, males and females were affected with a similar frequency.

LD patients were males in 60% of sporadic cases (Table 3) and in 71.9% of outbreaks, considering only the events reporting gender distribution. The median age was 56.5 years (range: 10–88) in sporadic cases and over 60 years in nine of the 13 LD outbreaks in which the age data was reported. Patient risk factors and underlying medical conditions were specified in 24 of the 34 LD events (71.3%), for a total of 155 cases. Figure 2 shows the occurrence of contributing factors and underlying medical conditions in these patients. Heavy smoking was the most frequent risk factor (58.7% of patients) and, among the underlying medical conditions, cardiovascular diseases (23.9%) and diabetes (11.0%) had the highest prevalence. Four cases of *Legionella* pneumonia occurred after near drowning, one in estuarine water and three in hot spring spas and public baths.

### 3.6. Environmental Contributing Factors

**Figure 3:**
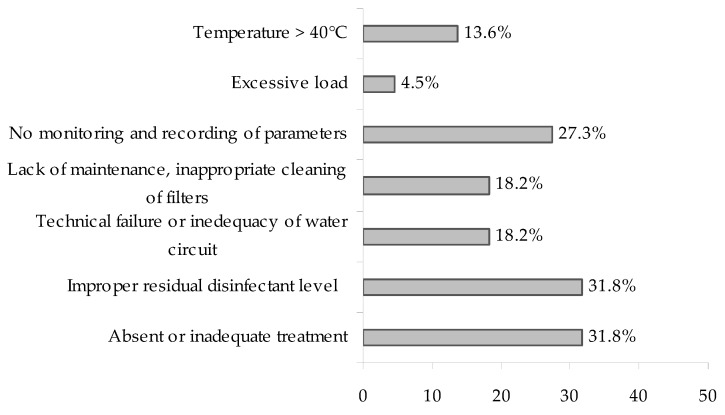
Distribution of environmental contributing factors in 22 recreational facilities associated with legionellosis events.

Excluding the only sporadic case related to estuarine water, environmental contributing factors were investigated in 22 out of 41 events. In only one of these, no contributing environmental conditions were found. In the other 21 events, inadequate water treatment and residual disinfectant below the recommended levels were the most frequent factors that could have favoured the onset of cases or outbreaks. The water temperature was reported in only four events and in three of these the temperature was above 40 °C (Figure 3). In PF events, the most frequent environmental contributing factors were those related to plant maintenance and chemical treatment management (i.e., inappropriate residual disinfectant concentration), while the inadequacy or absence of the treatment system was observed only for LD cases or outbreaks. This could be explained by the fact that many LD events occurred in private hot tubs not subjected to a supplementary disinfection system. 

*Legionella* spp. were isolated from the environmental samples of 32 facilities, at concentrations higher than 10^3^ cfu/L in water samples obtained from 11 of them (34.4%).

## 4. Discussion

This review aimed to evaluate the cases and outbreaks of legionellosis associated with exposure to recreational water since the disease was first described in 1976. Both sporadic cases and outbreaks of LD and PF, described in the scientific literature, were included. Relevant findings from 47 articles were synthesized, including 42 legionellosis events (17 sporadic cases and 25 outbreaks).

### 4.1. Temporal and Geographical Distribution

The events of legionellosis correlated with exposure to recreational water showed a non-homogeneous distribution over time. In the 1980s, only four events were reported, probably because, in these first years, there was a lower awareness of the problem and many cases were not identified or associated with exposure to recreational water. In the 1990s and 2000s, the number increased (16 and 19 events, respectively) and then declined in the years from 2010 until today (only three reported in the literature). It could be hypothesized that the increase in knowledge and awareness of risks associated with recreational water led to an improvement in the management and maintenance and control measures, also after the issuing of international guidelines on the control of legionellosis in recreational facilities. In 2006, the WHO Guidelines for safe recreational water environments recommended the implementation of safety plans and adequate control measures in pools and hot tubs [17]. Moreover, from 2005, the European Legionnaires’ Disease Surveillance Network (ELDSNet, previously EWGLI), with respect to *Legionella* risk reduction in whirlpool spas, recommended continuous treatment with 2–3 mg/L of chlorine or bromine, the checking of these levels almost three times a day, the replacement of at least half of the water each day, sand filters backwashed daily, and cleaning and disinfection of the whole system every day [66]. The implementation of these measures could explain the reduction in the number of events in the most recent period.

The reported events of legionellosis involved 10 countries, with the highest number of events (18) and cases (385) in Japan, where the habit of frequenting hot spring spas and public baths is very widespread, following a long-established tradition in Japanese culture. Moreover, the average water temperature in hot tubs in Japan usually ranges from 40 °C to 43 °C, which is higher than in Europe (30–40 °C) [27].

### 4.2. Clinical Features and Laboratory Evidence

This review includes both PF and LD events. PF cases totalled 620, only one of which was sporadic, the others being included in 14 outbreaks. The number of PF cases related to recreational water is probably underestimated: The benign nature of the disease, which often presents as an influenza-like illness, means that the cases, especially when sporadic, are not identified as legionellosis and are, therefore, not subjected to laboratory diagnosis. In the selected PF events, laboratory diagnosis was performed only in outbreaks, and *Legionella* spp. were never culturally isolated. On the contrary, in the events involving LD cases, cultural isolation from patients’ specimens allowed the species to be identified in 75% of the sporadic cases and in 11 of the 18 outbreaks with LD cases (61.1%). 

Among the different species and serogroups, *L. pneumophila* SG 1 (three sporadic cases and 15 outbreaks of LD) and SG 6 (five sporadic cases and two outbreaks of LD) were the agents most frequently responsible, while, among the other species, *L. micdadei* was implicated in three outbreaks of PF and two outbreaks of mixed PF and LD. In five events, various species or serogroups were involved [27,30,42,58,62], one of which was the first case where the same genotype of *L. rubrilucens* was isolated from the LD patient’s sputum and the hot spring water [30].

This review confirms certain known characteristics of the epidemiology of legionellosis. PF cases showed no evidence of underlying risk factors and PF outbreaks had a high attack rate, with no difference between males and females. On the contrary, LD cases prevalently involved males and individuals presenting risk factors, such as smoking and all the underlying medical conditions that reduce immune defenses. In LD outbreaks, the attack rate is low and the fatality rate is high (on average, 6.3%, but up to 31.2% in events related to private hot tubs).

### 4.3. Recreational Water Facilities and Risk Assessment

Most events occurred in public spa pools (22 events, 744 cases). Of these, 10 were associated with hotels or similar residential facilities and, therefore, fall within the surveillance system for legionellosis linked to travel, which in Europe is carried out by the ELDSNet and coordinated by ECDC. The recreational facilities supplied by natural water (hot spring, thermal water) were the setting for 12 events, 10 of which with a single case. Most studies referring to hot spring/thermal spas (seven out of 11) did not specify if the water was treated or untreated and how the facility was managed; this is a limitation that makes it difficult to draw conclusions about the environmental conditions contributing to these infections.

The recommended standards for *Legionella* spp. in hot tub water range from 0/100 mL to 1000/L in different countries [67]. In the selected studies, the environmental isolates of *Legionella* spp. are reported in 32 events, but only 13 specify the level of contamination, which ranges between 100 cfu/L and >10^6^ cfu/L. However, it should be noted that the isolation of *Legionella* spp. from environmental samples was carried out after the legionellosis event had occurred and so the environmental conditions may have changed. The lack of data on the *Legionella* concentrations in the water, and on the frequency and duration of exposure, makes it difficult to perform a risk assessment. Various studies tried to estimate the risk for *Legionella* infection due to spa pool use. Bouwknegt et al., (2013) estimated that the infection risk for sitting in an active whirlpool for 15 min ranged from around 3% for a concentration of 10 *L. pneumophila* cfu/L to up to 95% for >1000 cfu/L [68]. These findings suggest that a risk cannot be excluded even in the presence of very low concentrations, and stricter requirements may be needed to ensure adequate protection for users. Azima et al. (2013) suggested a reference value of <1 cfu/L, which is less than the current detection limit [69].

### 4.4. Epidemiological Investigation and Strength of Evidence

The epidemiological investigation included an analytical study in nine outbreaks, four with a case-control study and five with a retrospective cohort study. In all the events related to private hot tubs, only descriptive epidemiology was carried out. This is justified by the difficulty in such events to find a control group not exposed to the private hot tub. Also, sporadic cases were studied only through descriptive epidemiology (case reports).

The environmental investigation was often delayed with respect to the event onset and, in some cases, was made after control measures had already been adopted. These measures are specified only in a limited number of articles and information is lacking on the follow-up procedures in almost all the articles. Many studies do not report the environmental conditions that could have favoured such infections. In 19 events, no information is available on the type of water treatment, the level of residual disinfectant, or the state of maintenance of the facility. Only in three events is the water temperature specified, a factor that, in these types of recreational facilities, plays a fundamental role in the development of *Legionella* spp. and was probably co-responsible for three LD cases associated with near drowning in hot spring spas and public baths [32,35,37]. Lying in or sitting up to the neck in hot water (above 40 °C), especially in combination with alcohol consumption, may cause drowsiness, which may then lead to unconsciousness and, consequently, drowning [70].

Based on the selected criteria, the strength of evidence linking the cases/outbreaks to the recreational water facilities was strong in 52.4% of events, probable in 21.4%, and possible in 23.9%. Strong evidence was principally attributable to the results of analytical study in nine events, and to the match of environmental and clinical isolates in 17 events. The comparison between strains of environmental and clinical origin using molecular biology techniques was carried out at a very high level of frequency, especially in cases concerning LD (43.7% of sporadic cases and 81.8% of LD outbreaks).

### 4.5. Limitations

The present study was limited to articles published in English or with an exhaustive abstract in English, and only peer-reviewed literature was considered. Furthermore, the legionellosis events that are published represent only part of the overall number of cases: Larger LD outbreaks are more likely to be published than sporadic cases and smaller events, especially of Pontiac fever. Also, the review does not include cruise ship cases [18] and cases associated with display spa pools in retail premises, fairs, exhibitions, and shows [71,72], which represent another important source of infection. Therefore, the role of the recreational facilities as a source of infection is underestimated, also considering that in many LD and PF cases the source of *Legionella* remains unknown [3,16].

The heterogeneity of epidemiological investigations, in terms of study design, sample size, and information about the duration of exposure and environmental contributing factors, limited the comparison of results. In particular, the lack of information about the treatment and management of recreational facilities makes it difficult to exhaustively evaluate the role of environmental conditions.

## 5. Conclusions

Data extracted from the articles in this systematic review show that hot tubs, whirlpools, and spa pools represent an important source of infection of *Legionella* spp., given the number of cases involved (1079 from 1981 to 2015), the number of deaths (29), and the high percentage of events with strong evidence of an association. On the contrary, the risk related to the natural recreational water of rivers and lakes appears negligible: The only sporadic case reported is a case consequent to a near-drowning in estuarine water [20].

Among the cases included in this review, PF cases were the most numerous and were caused by a variety of species and serogroups: *L. pneumopghila* SG 6 and *L. micdadei* were the most often responsible agents, while *L. pneumophila* SG 1 was responsible for most LD cases. Unlike PF cases, LD cases prevalently involved individuals presenting risk factors, such as smoking, and underlying medical conditions that reduce immune defenses.

Certain operating conditions that facilitate the formation of aerosol, such as the high temperature of the water and the presence of hydromassage systems, are risk factors inherent to this kind of recreational water. In hot tubs and similar facilities, it is impractical to maintain a water temperature outside the range considered at risk. Therefore, other management strategies need to be implemented, which may include appropriate design and adequate disinfection residual and proper maintenance and cleaning of equipment as well as adequate ventilation. Features, such as water sprays, should be periodically cleaned and flushed with a level of disinfectant adequate to eliminate *Legionella* spp. [3,17,67]. In this review, the environmental conditions were described for 22 events, and in 21 of these (95.5%) at least one of the preventive measures recommended by the various guidelines was not respected. Therefore, it seems important to increase collaboration between the different professionals involved (public health experts, policy makers, facility managers, technical staff, equipment manufacturers) to improve the knowledge of the operators and their awareness of the risk and to favour compliance with control measures.

## Figures and Tables

**Table 1:** Strength of evidence linking a case/outbreak of legionellosis with a recreational water system.

Strength of Evidence	Epidemiological and Microbiological Criteria
Strong	An analytical epidemiological study demonstrates a significant association between case/outbreak of legionellosis and exposure to the recreational water; and the same species and serogroups of *Legionella* spp. are isolated from the water system at any concentration.
	Or
	Descriptive epidemiology suggests that the case/outbreak is related to the recreational water and excludes obvious alternative explanations; and *Legionella* spp. are isolated from the water system at any concentration and environmental isolates show identical genotype profiles of clinical isolates.
Probable	An analytical epidemiological study demonstrates a significant association between case/outbreak of legionellosis and exposure to the recreational water; and *Legionella* spp. are not isolated from the recreational water.
	Or
	Descriptive epidemiology suggests that the case/outbreak is related to the recreational water and excludes obvious alternative explanations; and the same species and serogroups of *Legionella* spp. are isolated from the water system at any concentration.
Possible	Descriptive epidemiology suggests that the case/outbreak is related to exposure to the recreational water and excludes obvious alternative explanations; and *Legionella* spp. are not isolated from the recreational water.

**Table 2:** Events of Pontiac fever (PF) and Legionnaires’ disease (LD) associated with recreational water.

Characteristics of the Events	Hot Tub/Whirlpool/Japanese Bath in Private House (8 Events)	Spa Pools/Public Baths in Public Centres or Hotels (22 Events)	Hot Spring Spa, Thermal Spa, Recreational Surface Water (12 Events)	Total Recreational Waters (42 Events)
Number of events with single cases	5	2	10	17
Number of outbreaks or events with repeated cases ^a^	3	20	2	25
Number of total cases	28	744	307	1079
Median number of cases per outbreak (range)	6 (4–13)	23.5 (3–170)	148.5 (2–295)	23 (2–295)
Total number of PF cases (fatal cases)	22 (0)	598 (0)	0	620 (0)
Total number of LD cases (fatal cases)	6 (1)	146 (16)	307 (12)	459 (29)
Fatality rate on total cases (on LD cases)	3.6% (16.7%)	2.2% (11.0%)	3.9% (3.9%)	2.7% (6.3%)
Analytical epidemiology in outbreak investigation (% of total outbreaks)	0 (0%)	8 (40.0%)	1 (50.0%)	9 (36.0%)
Events with environmental investigation (% of total events)	6 (75.0%)	20 (90.9%)	9 (75.0%)	35 (83.3%)
*Legionella* spp. detected in environmental water samples (% of total events)	4 (50.0%)	20 (90.9%)	8 (66.7%)	32 (76.2%)
Identical *Legionella* genotype in clinical and environmental isolates (% of total events)	1 (12.5%)	6 (27.3%)	7 (58.3%)	14 (33.3%)
Strength of evidence				
Strong (%)	1 (12.5%)	15 (68.2%)	7 (58.3%)	23 (52.4%)
Probable (%)	3 (37.5%)	5 (22.7%)	1 (8.3%)	9 (21.4%)
Possible (%)	4 (50.0%)	2 (9.1%)	4 (33.3%)	10 (23.9%)

^a^ 22 outbreaks and three events with repeated cases or cluster.

**Table 3:** Events with sporadic cases of Pontiac fever (PF) and Legionnaires’ disease (LD) associated with recreational water.

	Pontiac Fever (1 Event) ^a^	Legionnaires’ Disease (16 Events) ^b^
Number of cases (fatal cases)	1 (0)	16 (5)
Gender		
Males		9
Females		6
Not reported	1	1
Median age (range)	37	56.5 (10–88)
Confirmation by culture in clinical specimen	0	12 (75.0%)
*Legionella* species and serogroup		
*L. pneumophila* SG 1	0	3 (18.7%)
*L. pneumophila* SG 2	0	1 (6.2%)
*L. pneumophila* SG 3	0	2 (12.5%)
*L. pneumophila* SG 4	0	1 (6.2%)
*L. pneumophila* SG 6	0	5 (31.2%)
*L. pneumophila* SG 13	0	2 (12.5%)
*L. pneumophila* (SG not reported)	1 (100%)	1 (6.2%)
*L. rubrilucens*	0	1 (6.2%)
Environmental source		
Private hot tub	1	4 (25.0%)
Public and hotel spa	0	2 (12.5%)
Hot spring/thermal spa	0	9 (56.2%)
Estuarine water	0	1 (6.2%)
*Legionella* colonization		
<1000 cfu/L	0	2 (12.5%)
1000–10,000 cfu/L	0	2 (12.5%)
>10,000 cfu/L	0	2 (12.5%)
Not reported	1 (100%)	11 (68.7%)
Identical *Legionella* genotype in clinical and environmental isolates	0	7 (43.7%)
Strength of evidence		
Strong (%)	0	7 (43.7%)
Probable (%)	1 (100%)	2 (12.5%)
Possible (%)	0	7 (43.7%)

^a^ [23]; ^b^ [20,24,25,26,27,28,29,30,31,32,33,34,35,36,37,38].

**Table 4:** Outbreaks of Pontiac fever (PF) associated with recreational water.

Event No. Country, Year (Reference)	Water System	*Legionella* spp. (Confirmed Diagnosis Based on)	No. of Cases (Fatal Cases)	Attack Rate	Proportion of Males	Median Age (Range)	Environmental Isolates (cfu/L)	Strength of Evidence
1 Vermont, US, 1981 [39]	Inn whirlpool spa	*L. pneumophila* SG 6 (antibody titre)	34 (0)	45.9%	53.0%	27.9	*L. pneumophila* SG 1,6 *L. dumoffii*	Strong
2 Michigan, US, 1982 [40]	Public whirlpool spa (women’s pool)	*L. pneumophila* SG 6 (antibody titre)	14 (0)	29.8%	0	32 (25–39)	*L. pneumophila* SG 6	Strong
3 Colorado, US, 1992 [41]	Resort indoor whirlpool	*L. pneumophila* SG 6 (antibody titre)	13 (0)	38.0%	na	na	*L. pneumophila* SG 6 (>1,000,000)	Strong
4 Denmark, 1995 [42]	Private summerhouse whirlpool	*L. pneumophila* SG 1 (culture, antibody titre) *L. micdadei* (antibody titre)	13 (0)	86.7%	na	na	negative samples (after whirlpool cleaning)	Possible
5 Wisconsin, US, 1998 [43]	Hotel whirlpool spa	*L. micdadei* (antibody titre)	45 (0)	whirlpool area: 66.0% whirlpool users: 71.0%	na	na	*L. micdadei* (90,000/L)	Strong
6 Sweden, 1999 [44]	Hotel whirlpool spa	*L. micdadei* (antibody titre)	29 (0)	whirlpool area: 71.0% whirlpool users: 88.9%	37.9%	41 (21–57)	negative samples	Probable
7 England, 2008 [45]	Resort whirlpool spa	*L. pneumophila* SG 1 (antibody titre, urinary antigen)	6 (0)	86.0%	0	(24–37)	*Legionella* non *pneumophila* (100/L)	Probable

na: Not available; clinical and environmental isolates were never compared by molecular typing.

**Table 5:** Outbreaks of Legionnaires’ disease (LD) associated with recreational water.

Event No. Country, Year (Reference)	Water System	*Legionella* spp. (Diagnosis Based on)	Number of Cases (Fatal Cases)	Attack Rate	Proportion of Males	Median Age (Range)	Environmental Isolates (cfu/L)	Strength of Evidence
1 Vermont, US, 1987 [46]	Inn whirlpool spa	*L. pneumophila* SG 1 (culture, antibody titre)	3 (0)	na	na	na	*L. pneumophila* SG 1,4	Strong
2 Netherlands 1992–96 [47]	Public spa sauna’s footbath	*L. pneumophila* SG 1 (culture)	6 repeated cases (2)	na	83.3%	males: 50 females: 28	*L. pneumophila* SG 1	Strong
3 France 1994–97 [21]	Thermal spa	*L. pneumophila* SG 1 (culture)	2 repeated cases (1)	na	50%	54.5 (40–69)	*L. pneumophila* SG 1,2,3,6,9,13 *L. dumoffii*	Strong
4 Japan, 1996 [48]	Public Japanese spa	*L. pneumophila* SG 1 (antibody titre)	3 (0)	na	na	na	*L. pneumophila* SG 1	Probable
5 Japan, 2000 [27]	Public bath house	*L. pneumophila* SG 1,6 (culture, antibody titre, urinary antigen)	23 (2)	0.13%	91.3%	67 (50–86)	*L. pneumophila* SG 1 (880,000)	Strong
6 Japan, 2000 [49,50]	Public bath house	*L. pneumophila* SG 1 (culture, antibody titre, urinary antigen)	34 (20 confirmed) (3)	0.20%	65.0% (only confirmed)	62.2 (27–85)	*L. pneumophila* SG 1,3,5,6 (11400–84200)	Strong
7 Japan, 2002 [22,51,52,53,54,55]	Hot spring bath	*L. pneumophila* SG 1 (culture, antibody titre, urinary antigen)	295 including suspected cases (7)	1.5%	64.5% (of 76 examined)	65 (9–95)	*L. pneumophila* SG 1,8 (1,600,000) *L. dumoffii* (5,200,000) *L. londiniensis* (15,000,000)	Strong
8 Japan, 2003 [27]	Public bath house	*L. pneumophila* SG 1 (culture)	9 (1)	0.13%	na	65 (52–82)	*L. pneumophila* SG 1 (1,300,000)	Probable
9 France, 2010 [56]	Public whirlpool spa	*L. pneumophila* SG 1 (culture, urinary antigen)	3 (1)	na	33.3%	50 (30–70)	*L. pneumophila* SG 1 (150,000)	Strong
10 Spain, 2011–12 [57]	Hotel spa pool	*L. pneumophila* SG 1 (culture)	Total: 44 (6) Cluster1: 21 Cluster2: 2 Cluster3: 3 Cluster4: 18	na	na	tourists: 71.5 hotel workers: 49.5	*L. pneumophila* SG 1 *L. micdadei*	Strong
11 Japan, 2015 [58]	Spa house (men’s pool)	*L. pneumophila* SG 1,13 (culture)	7 (0)	na	100%	66.3	*L. pneumophila* SG 1,13	Strong

na: Not available; clinical and environmental isolates showed correlated molecular profiles in events No. 1, 2, 3, 5, 6, 7, 9, 10, and 11.

**Table 6:** Outbreaks of Pontiac fever (PF)/Legionnaires’ disease (LD) associated with recreational water.

Event No. Country, Year (Reference)	Water System	*Legionella* spp. (Diagnosis Based on)	Number of Cases PF + LD (Fatal Cases)	Attack Rate	Proportion of Males	Median Age (Range)	Environmental Isolates (cfu/L)	Strength of Evidence
1 Scotland, 1987–88 [19,59]	Hotel whirlpool spa	*L. micdadei* (antibody titre)	169 + 1 (0)	90.9% (LD: 0.5%)	48.8%	32 (2–72)	*L. micdadei*	Probable
2 Vermont US, 1991 [60]	Private hot tub in holiday home	*L. pneumophila* SG 1 (antibody titre)	5 + 1 (0)	na	na	na	not investigated	Possible
3 Georgia US, 1999 [61]	Hotel whirlpool spa	*L. pneumophila* SG 6 (culture, antibody titre, urinary antigen)	22 + 2 (0)	22.0% (LD: 1.8%)	na	PF: 12 (5–31) LD: 66 (61–71)	*L. pneumophila* SG 6	Strong
4 Illinois US, 2002 [62]	Hotel spa area	*L. micdadei* *L. maceachernii* (antibody titre)	49 + 1 (0)	62.7% (LD: 1.2%)	46%	20 (2–58)	*L. micdadei* *L. maceachernii* *L. dumoffii*	Strong
5 Oklaoma US, 2004 [63]	Hotel pool and hot tub area	*L. pneumophila* SG 1 (antibody titre, urinary antigen)	101 + 6 (0)	33.7% (LD: 1.9%)	PF: 43.6% LD: 100%	PF: 15 (2–65) LD: 6.5 (2–44)	*L. pneumophila* SG 1	Strong
6 England, 2006 [64]	Leisure club spa pool	*L. pneumophila* SG 1 (antibody titre, urinary antigen)	116 + 2 (0)	na	PF: 41.4% LD: 100%	(18–85)	*L. pneumophila* SG 1	Probable
7 Netherlands, 2009 [65]	Private outdoor whirlpool spa	*L. pneumophila* SG 1 (antibody titre, urinary antigen)	3 + 1 (1 LD)	na	PF: 66.7% LD: 0%)	PF: 54 (52–83) LD: 78	*L. pneumophila* SG 1	Probable

na: Not available; clinical and environmental isolates showed correlated molecular profiles in the event No. 3.

## Abbreviations

The following abbreviations are used in this manuscript:

LD	Legionnaires’ Disease
PF	Pontiac Fever
cfu	colony forming unit
